# Impact of lesion size and localization on symptom severity in intestinal endometriosis

**DOI:** 10.3389/fmed.2026.1760665

**Published:** 2026-02-12

**Authors:** Yuliia Ocheretna, Natalia Rozhkovska, Vitaliy Kozhakov, Vasyl Gladchuk, Hanna Shytova, Yurii Petrovskiy, Igor Gladchuk, Harald Krentel

**Affiliations:** 1 Department of Gynecology and Obstetrics, Odesa National Medical University, Odesa, Ukraine; 2 Department of Gynecology and Obstetrics, Klinikum Aschaffenburg-Alzenau, Aschaffenburg, Germany

**Keywords:** #ENZIAN classification, deep endometriosis, dyschezia, gastrointestinal symptoms, intestinal endometriosis, laparoscopic surgery

## Abstract

**Background/objectives:**

Intestinal endometriosis (IE) often presents with dyschezia, gastrointestinal (GI) dysfunction, dyspareunia, and dysmenorrhea. The impact of lesion localization and size on the severity of symptoms remains insufficiently studied, complicating preoperative diagnosis, treatment planning, and surgical excision. The aim is to assess preoperative symptoms in women with intestinal endometriosis and to evaluate the impact of lesion localization and size according to the #Enzian classification on the severity of pain and GI symptoms.

**Methods:**

A retrospective analysis of medical records was conducted for 135 patients with laparoscopically confirmed intestinal endometriosis (IE) at the Multidisciplinary Medical Center of Odesa National Medical University. Lesion localization and size were classified intraoperatively according to the #Enzian classification in a surgically treated cohort of women. The presence and severity of pain (dysmenorrhea, dyspareunia, dyschezia) and gastrointestinal symptoms (constipation, diarrhea, abdominal pain, bloating, nausea, fecal incontinence, hematochezia) were analyzed.

**Results:**

Pain and/or gastrointestinal symptoms were present in 121 patients (89.6%), while 14 patients (10.4%) did not report such symptoms. Large rectal nodules (#Enzian C3) were associated with higher severity of dyschezia, hematochezia, and abdominal pain compared with smaller lesions (#Enzian C1–C2) (*p* < 0.001). Hematochezia was observed only in patients with #Enzian compartment C (C2, C3) involvement. Patients with additional bowel lesions (#Enzian FI) were associated with a higher risk of bloating compared with rectal lesions alone. Combined lesions (#Enzian C and FI) are also associated with more severe hematochezia compared with #Enzian C/#Enzian FI alone (*p* = 0.002) and dyschezia compared with #Enzian FI alone (*p* = 0.03). #Enzian FI lesion size did not influence these results significantly but the analysis was likely underpowered for FI size effects.

**Conclusion:**

Large rectal lesions (#Enzian C3) are associated with more severe dyschezia, abdominal pain, and hematochezia. The presence of additional intestinal lesions (#Enzian FI) is associated with increased severity of hematochezia and the risk of bloating.

## 1 Introduction

Intestinal endometriosis (IE) is characterized by the presence of deep endometriotic lesions within the intestinal wall, commonly affecting the rectosigmoid junction and the rectum (1, 2). This subtype of deep endometriosis (DE) occurs in approximately 5–12% of patients with endometriosis (3). Preoperative diagnosis of IE remains a complex and crucial task, as surgical management is associated with considerable risks and potential complications (4–7).

IE may manifest through a wide range of pain-related symptoms, including dyschezia, dyspareunia, and dysmenorrhea, as well as various gastrointestinal symptoms (8–12).

The colorectal manifestations of IE are often compared with those observed in patients after low anterior resection (LARS-like symptoms) (13, 14). Among symptomatic women with deep intestinal endometriosis, a possible association between the localization and size of lesions and the presence or severity of symptoms has been discussed (15–17). Understanding such associations may be useful for improving preoperative diagnosis and the development of tailored treatment strategies.

However, intestinal lesions may also be present in patients who do not report the above-mentioned symptoms. The aim of this study was to evaluate preoperative symptoms in women with IE and to determine the potential influence of lesion localization and size on the severity of symptoms according to the #Enzian classification.

## 2 Materials and methods

A retrospective analysis of medical records was performed for 135 patients with surgically verified IE. All surgical procedures were performed by a single expert surgeon with a multidisciplinary team (coloproctologists and urologists) at the Multidisciplinary Medical Center of Odesa National Medical University between 2019 and 2023. The main indications for surgery were pain resistant to conservative treatment, severe GI disorders, and/or infertility. The primary surgical techniques included rectal shaving and nerve-sparing segmental bowel resection (NSBR) with end-to-end anastomosis. Indications for NSBR were multifocal lesions, lesions > 3 cm and significant organ dysfunction (severe dyschezia, severe rectal bleeding or intestinal obstruction), and nodules located above the rectosigmoid junction (#Enzian compartment FI). Inclusion criteria were age ≥18 years old and the absence of inflammatory bowel disease or suspected colorectal malignancy.

All patients (*n* = 135, 100%) underwent complete preoperative assessment, including anamnesis, general clinical examination, symptom evaluation using structured questionnaires, bimanual gynecologic examination, and transvaginal ultrasound according to the International Deep Endometriosis Analysis (IDEA) criteria. When necessary, magnetic resonance imaging (MRI), tumor marker assessment, abdominal ultrasound, colonoscopy, barium enema, and computed tomography (CT) of the bladder and ureters were performed.

Baseline clinical and demographic characteristics (age, body mass index, parity, and history of previous surgical treatment) were summarized using descriptive statistics. Symptom evaluation was performed during the standardized preoperative assessment, after the indication for surgical treatment had been established and prior to surgery by treating physician. Pain-related symptoms (dysmenorrhea, dyspareunia, and dyschezia) were assessed using the Visual Analogue Scale (VAS) ranging from 0 to 10, where “0” indicated absence of the symptom and “10” represented maximum pain severity. Dysmenorrhea with a score ≤ 3 points was considered a nonspecific symptom for deep endometriosis, including intestinal endometriosis. This cohort may be considered mildly symptomatic or asymptomatic. Gastrointestinal symptoms (constipation, diarrhea, intestinal pain, bloating, nausea, fecal incontinence, blood in stool) were evaluated using the Verbal Rating Scale (VRS) from 0 to 4, where “0” indicated absence of symptoms and “4” indicated the most severe manifestation. The assessment reflected the presence and severity of symptoms at least over the past 4 weeks. Symptom frequency was defined as the number of patients reporting each symptom shown as absolute values (n) and percentages (%). All data were available for all included patients. Lesion measurements were performed intraoperatively by expert surgeon using a laparoscopic instrument with a diameter of 5 mm. After excision, the specimen was measured postoperatively using a measuring tape. Intestinal lesions were classified as #Enzian C (rectum) and #Enzian FI (other intestinal locations) according to the #Enzian classification. Size-based stratification was as follows: #Enzian C1 includes rectal lesions below 1 cm in longitudinal diameter; #Enzian C2 lesions from 1 to 3 cm; and #Enzian C3 lesions > 3 cm. Intestinal lesions proximal to the rectosigmoid junction (> 16 cm from anal verge) were classified as #Enzian FI (18). Size of #Enzian FI lesion is not yet defined in the current #Enzian classification system. We included size of #Enzian FI lesions in analogy to #Enzian C. In cases of multiple rectal lesions, the index lesion was defined as the largest lesion. In cases of confluent lesions, total extent of lesions was considered for grading. Histological confirmation of intestinal endometriosis was obtained routinely in all cases.

Statistical analysis was performed using the R software environment (version 4.3.2; R Foundation for Statistical Computing, Vienna, Austria). For quantitative variables, the mean, standard deviation (SD), standard error (SE), minimum and maximum values, and 95% confidence intervals (95% CI) were calculated. The normality of data distribution was assessed using the Shapiro–Wilk test, and the homogeneity of variances was evaluated using Levene’s test. Group comparisons were conducted using ANOVA followed by *post hoc* testing. In cases of non-normal data distribution, the nonparametric Kruskal–Wallis test with post hoc pairwise comparisons (Dunn’s test with Bonferroni correction) and the Mann–Whitney U test were applied. Categorical variables were compared using Pearson’s chi-square test (χ^2^). A *p*-value < 0.05 was considered statistically significant. Comparisons of symptom severity in relation to #Enzian compartment C lesion size and #Enzian FI lesion size were consider as pre-specified analyses.

## 3 Results

420 patients underwent surgery for endometriosis at the Multidisciplinary Medical Center of Odesa National Medical University from 2019 to 2023. From this cohort, patients with surgically and histologically confirmed intestinal involvement (n = 135) were selected. All patients provided preoperative consent for data processing in clinical research. All identified patients fulfilled the predefined inclusion criteria (age ≥18 years, the absence of inflammatory bowel disease or suspected colorectal malignancy).

The baseline characteristics of all patients are summarized in Table 1.

**Table 1:** Baseline characteristics of the patients (*n* = 135).

Parameter	Value
Age, years (M ± SD)	34.1 ± 6.5
Mean BMI, kg/m^2^ (M ± SD)	22.4 ± 2.9
Hormonal therapy for endometriosis, n (%)	44 (32.6)
Previous surgery for endometriosis, n (%)	34 (25.2)
Gravidity 0, n (%)	83 (61.5)
Gravidity ≥1, n (%)	52 (38.5)
Nulliparous, n (%)	103 (76.3)
Parity ≥1, n (%)	32 (23.7)

Data are presented as mean ± SD for continuous variables and as percents for categorical variables. Percentages are calculated using the total cohort as denominator (*n* = 135). BMI—body mass index; SD—standard deviation.

#Enzian C lesions were detected in 113 patients (83.7%), while lesions in other intestinal localizations (#Enzian FI) were identified in 33 patients (24.4%). Among all patients, small rectal nodules (#Enzian C1) were observed in 28 cases, #Enzian C2 in 61 cases, and large rectal nodules (#Enzian C3) in 24 patients. The anatomical distribution of #Enzian FI lesions is presented in Table 2.

**Table 2:** Distribution of #Enzian compartment FI lesions: single (*n* = 22), combined (*n* = 11), total (*n* = 33).

Site	Single intestinal lesions (%), *n* = 22	Combined (%), *n* = 11	Total (%), *n* = 33
Sigmoid colon	10 (45.5)	8 (72.7)	19 (57.6)
Caecum	4 (18.2)	1 (9.1)	5 (15.2)
Appendix	4 (18.2)	1 (9.1)	5 (15.2)
Transverse colon	3 (13.6)	1 (9.1)	3 (9.1)
Small intestine	1 (4.5)	0 (0)	1 (3.0)

FI describes involving other intestinal structures besides the rectum: lesions cranial to the rectosigmoid junction (above 16 cm from the anus - sigmoid, transverse colon, caecum, appendix, and small bowel) (18). Percentages are calculated within each column using the respective subgroup denominator (single lesions, combined lesions, total cases of FI #Enzian involvement).

The distribution by lesion size in the FI compartment was as follows: 19 patients had nodules < 1 cm, lesions 1–3 cm were detected in 12 cases, and large nodules (> 3 cm) were detected in 2 cases. Combined involvement of compartments C and FI was observed in 11 patients (8.1%). Among patients with combined lesions, C1 was observed in 2 cases, C2 in 6 cases, and C3 in 3 cases. IE was associated with additional peritoneal, ovarian endometriosis, deep endometriosis or adenomyosis in 94.8% of cases. Isolated intestinal lesions were observed in only 5.2% of patients (19).

The severity of pain-related symptoms was assessed in all 135 patients (100%). Table 3 represents the distribution and severity of pain symptoms assessed by VAS. A total of 121 patients (89.6%) presented with pain and/or gastrointestinal symptoms.

**Table 3:** Evaluation of preoperative pain-related symptoms using the VAS.

Symptom	Patients, *n* (%)	Preoperative VAS score (M ± SD)
Dysmenorrhea	135 (100)	6.14 ± 1.95
Dyspareunia	98 (72.6)	5.57 ± 2.89
Dyschezia	76 (55.6)	5.44 ± 2.92

Percentages represent symptom frequency in the total cohort (*n* = 135). VAS scores are presented as mean ± SD calculated among symptomatic patients (VAS>0). VAS – Visual Analogue Scale; M – mean; SD – standard deviation.

The severity of dyschezia was significantly higher in patients with C3 lesions compared with C1 and C2 with large effect size (η^2^_h_ = 0.148, 95% CI 0.04–0.32, *p* < 0.001). The severity of dysmenorrhea and dyspareunia did not differ significantly with increasing size of the rectal nodule (*p* = 0.452 and *p* = 0.052, respectively) (Table 4).

**Table 4:** Comparison of pain-related symptom severity assessed by VAS according to #Enzian C involvement (no C involvement, C1, C2, C3).

Symptom	None (*n* = 22)	C1 (*n* = 28)	C2 (*n* = 61)	C3 (*n* = 24)	*p* value
Dyschezia	0 (0–4)	2 (0–5)	4 (0–5)	7 (1.5–8)	<0.001^1^
Dysmenorrhea	6 (5-8)	6 (5–8)	6 (6–7)	7.5 (5–8)	0.452
Dyspareunia	4 (0–6)	5 (0–6)	5 (2–6)	6 (4.75–8)	0.052

Data are presented as median (interquartile range). Preoperative pain-related symptoms were assessed using the Visual Analogue Scale (VAS, 0–10; 0 = no symptoms, 10 = maximal severity). Post hoc Dunn–Bonferroni correction was applied for pairwise comparisons.

^1^C2-C3 *p* = 0.001; C1-C3 *p* < 0.001; C3- no C involvement *p* < 0.001.

Abdominal pain and hematochezia severity were higher in patients with C3 lesions compared with those with C1–C2 lesions, with large effect sizes (η^2^_h_ = 0.285, 95% CI 0.13–0.50, p < 0.001, and η^2^_h_ = 0.213, 95% CI 0.06–0.42, *p* < 0.001, respectively) (Table 5). The severity of bloating (*p* = 0.968), constipation (*p* = 0.244), diarrhea (*p* = 0.900), nausea (*p* = 0.417), and fecal incontinence (*p* = 0.107) showed no statistically significant variation across lesion sizes in compartment C.

**Table 5:** Comparison of gastrointestinal symptom severity assessed by VRS according to #Enzian C involvement (no C involvement, C1, C2, C3).

Symptom	None (*n* = 22)	C1 (*n* = 28)	C2 (*n* = 61)	C3 (*n* = 24)	*p* value
Hematochezia	0 (0–0)	0 (0–0)	0 (0–0)	0 (0–2)	<0.001^1^
Constipation	1.5 (0–3)	0 (0–2.25)	1 (0–2)	2 (0–3)	0.244
Diarrhea	0 (0–0.75)	0 (0–1)	0 (0–1)	0 (0–2)	0.9
Bloating	2 (1–2)	2 (0–2)	2 (0–3)	2 (0–3)	0.968
Nausea	0 (0–0)	0 (0–3)	0 (0–3)	0 (0–3.25)	0.417
Abdominal pain	0 (0–0)	0 (0–0)	0 (0–0)	2.5 (0–3)	<0.001^2^
Fecal incontinence	0 (0–0)	0 (0–0)	0 (0–0)	0 (0–1)	0.107

Data are presented as median (interquartile range). Gastrointestinal symptoms were assessed using the Verbal Rating Scale (VRS, 0–4; 0 = absence, 4 = maximum severity). Post hoc Dunn–Bonferroni correction was applied for pairwise comparisons.

^1^C1-C2 *p* < 0.001; C1-C3 *p* < 0.001; C3-no C involvement *p* < 0.001.

^2^Post hoc Dunn test with Bonferoni correction: C2 - C3 *p* < 0.001, C1-C3 *p* < 0.001, C3 - no C involvement *p* < 0.001.

The severity of dysmenorrhea (*p* = 0.329), dyspareunia (*p* = 0.949), dyschezia (*p* = 0.629), hematochezia (*p* = 0.673), bloating (*p* = 0.865), constipation (*p* = 0.490), diarrhea (*p* = 0.942), intestinal pain (*p* = 0.807), nausea (*p* = 0.69), and fecal incontinence (p = 0.9) did not show any statistically significant differences between nodule sizes in #Enzian FI (Tables 6, 7).

**Table 6:** Comparison of pain-related symptom severity assessed by VAS according to #Enzian FI involvement and lesion size (no FI involvement, FI < 1 cm, FI 1–3 cm, FI > 3 cm).

Symptom	None (*n* = 102)	FI < 1 cm (*n* = 19)	FI 1–3 cm (*n* = 12)	FI > 3 cm (*n* = 2)	*p* value
Dyschezia	4 (0–5)	0 (0–4.5)	0 (0–5)	4.5 (4.25–4.75)	0.629
Dysmenorrhea	6 (5–7)	7 (5–8)	6 (5.75–7.25)	7.5 (6.75–8.25)	0.329
Dyspareunia	5 (0–6)	5 (0–6.5)	4 (3.75–6)	5.5 (4.75–6.25)	0.949

Data are presented as median (interquartile range). Preoperative pain-related symptoms were assessed using the Visual Analogue Scale (VAS, 0–10; 0 = no symptoms, 10 = maximal severity).

**Table 7:** Comparison of gastrointestinal symptom severity assessed by VRS according to l #Enzian FI involvement and lesion size (no FI involvement, FI < 1 cm, FI 1–3 cm, FI > 3 cm).

Symptom	None (*n* = 102)	FI < 1 cm (*n* = 19)	FI 1–3 cm (*n* = 12)	FI > 3 cm (*n* = 2)	*p* value
Hematochezia	0 (0–0)	0 (0–0)	0 (0–0)	0 (0–0)	0.673
Constipation	1.5 (0–3)	0 (0–2.25)	1 (0–2)	2 (0–3)	0.244
Diarrhea	0 (0–1)	0 (0–1)	0 (0–2.25)	0.5 (0.25–0.75)	0.942
Bloating	2 (0–2.75)	2 (1–2)	2 (1–2)	2 (2–2)	0.865
Nausea	0 (0–3)	0 (0–1.5)	0.5 (0–3)	2 (1–3)	0.699
Abdominal pain	0 (0–0)	0 (0–0)	0 (0–0)	0 (0–0)	0.807
Fecal incontinence	0 (0–0)	0 (0–0)	0 (0–0)	0 (0–0)	0.9

Data are presented as median (interquartile range). Gastrointestinal (GI) symptoms were evaluated using the Verbal Rating Scale (VRS, 0–4; 0 = absence, 4 = maximum).

Patients with combined #Enzian C + FI lesions reported higher dyschezia scores compared with patients with #Enzian FI lesions alone with small effect-size (η^2^_h_ = 0.03 95% CI 0–0.13, *p* = 0.04), while no significant difference was observed between #Enzian C alone and combined #Enzian C + FI lesions (Table 8).

**Table 8:** Comparison of pain-related symptom severity assessed by VAS among patients with #Enzian Compartment C (*n* = 102), FI (*n* = 22), and combined C + FI (*n* = 11) involvement.

Symptom	#Enzian C (*n* = 102)	#Enzian FI (*n* = 22)	Combined #Enzian C + FI (*n* = 11)	*p*
Dyschezia	4 (0–5)	0 (0–4)	5 (0–6.5)	0.04^1^
Dysmenorrhea	5 (0–6)	4 (0–6)	5 (4–7.5)	0.30
Dyspareunia	6 (5–7)	6 (5–8)	8 (6.5–8.5)	0.10

Data are presented as median (interquartile range). Preoperative pain-related symptoms were assessed using the Visual Analogue Scale (VAS, 0–10; 0 = no symptoms, 10 = maximal severity). Post hoc Dunn–Bonferroni correction was applied for pairwise comparisons. ^1^C + FI>FI (*p* = 0.03), C-FI (*p* = 0.055), C- C + FI (*p* = 0.4).

The severity of hematochezia was higher in the group with combined #Enzian C and FI lesions with moderate effect (η_H_^2^ = 0.08, 95% CI 0–0.31, *p* < 0.001), while all other GI symptoms did not show significant differences in this subanalysis (Table 9).

**Table 9:** Comparison of gastrointestinal (GI) symptom severity between patients with lesions in compartments C (*n* = 102), FI (*n* = 22), and combined (C + FI, *n* = 11) according to the #Enzian classification.

Symptom	#Enzian C (*n* = 102)	#Enzian FI (*n* = 22)	Combined #Enzian C + FI (*n* = 11)	*p*
Bloating	2 (0–2.75)	2 (1–2)	2 (1–2.5)	0.628
Diarrhea	0 (0–1)	0 (0–0.75)	0 (0–1)	0.859
Constipation	1 (1–2)	1.5 (0–3)	2 (0.5–2.5)	0.291
Nausea	0 (0–3)	0 (0–0)	0 (0–4)	0.074
Abdominal pain	0 (0–0)	0 (0–0)	0 (0–2.5)	0.108
Hematochezia	0 (0–0)	0 (0–0)	0 (0–2)	*p* = 0.002 (C + FI > C/FI)
Fecal incontinence	0 (0–0)	0 (0–0)	0 (0–0)	0.651

Data are presented as median (interquartile range). Kruskal–Wallis test was used for intergroup comparison. Post hoc Dunn–Bonferroni correction was applied for pairwise comparisons. A statistically significant difference was observed only for hematochezia (*p* = 0.002), which was more severe in patients with combined C + FI involvement.

Our analysis revealed no significant differences between patients with #Enzian C1–C3 lesions and the reported frequency of dyschezia (*p* = 0.12), dyspareunia (*p* = 0.28), bloating (*p* = 0.13), constipation (*p* = 0.43), diarrhea (*p* = 0.96), nausea (*p* = 0.36), or fecal incontinence (*p* = 0.12). Only the frequency of abdominal pain increased with larger lesion size. Among all patients with #Enzian C involvement who reported abdominal pain, 51.5% had large nodules (C3), 30.3% had C2 lesions, and only 9% had C1 lesions (*p* < 0.001). Hematochezia was observed in patients with rectal lesions larger than 1 cm (C2) — 25% of cases — and predominantly in those with large nodules (C3), accounting for 75% of cases (*p* < 0.001).

The frequency of pain-related and gastrointestinal symptoms showed no significant variation in relation to the size of #Enzian FI lesions: dyspareunia (*p* = 0.64), dyschezia (*p* = 0.35), hematochezia (*p* = 0.69), constipation (*p* = 0.42), diarrhea (*p* = 0.94), abdominal pain (*p* = 0.73), nausea (*p* = 0.58), fecal incontinence (*p* = 0.87).

All patients with combined intestinal nodules (#Enzian C and FI) reported bloating (100%), those with #Enzian FI involvement in 95.4%, and patients with isolated #Enzian C lesions in 68.6%. This difference was statistically significant (*p* = 0.004). No significant differences were found in the frequency of other evaluated GI symptoms (Table 10), dyspareunia and dyschezia.

**Table 10:** The distribution of gastrointestinal (GI) symptoms in patients with IE.

Symptom	#Enzian C *n* = 102 (%)	#Enzian FI *n* = 22 (%)	#Enzian C + FI *n* = 11 (%)	*p*
Bloating	70 (68.6)	21 (95.5)	11 (100)	0.004
Diarrhea	31 (30.4)	6 (27.3)	4 (36.4)	0.86
Constipation	54 (52.9)	14 (63.6)	8 (72.7)	0.34
Nausea	39 (38.2)	5 (22.7)	7 (63.6)	0.072
Abdominal pain	25 (24.5)	3 (13.6)	5 (45.5)	0.134
Hematochezia	8 (7.8)	0 (0)	4 (36.4)	0.002
Fecal incontinence	16 (15.7)	2 (9.1)	2 (18.2)	0.69

Data are presented as absolute values with percentages within each subgroup. Pearson’s chi-square test was applied for group comparisons. *p* < 0.05 was considered statistically significant.

Fourteen patients (10.4%) did not report gastrointestinal symptoms (VRS = 0), dyschezia (VAS = 0), dyspareunia (VAS = 0). Severity of dysmenorrhea in this group were no higher than 3 points by VAS. Among these patients, #Enzian C lesions were observed in 11 cases (C1: 4 patients [3.0%]; C2: 6 patients [4.4%]; C3: 1 patient [0.7%]). #Enzian FI lesions were identified in 3 patients (2.2%): nodules < 1 cm in 2 cases (1.5%) and nodules 1–3 cm in 1 patient (0.7%).

## 4 Discussion

Intestinal endometriosis represents one of the most complicated forms of the disease. It is associated with a wide range of symptoms, including severe pain and functional bowel disorders. Management of IE is challenging and requires adequate decision-making strategies. Recent publications emphasize the necessity of a multidisciplinary approach to diagnosis and management, as the choice between conservative and surgical treatment should be highly individualized depending on symptoms, reproductive goals, and disease extent, with careful evaluation of risks and benefits (20).

Symptom evaluation using diaries and questionnaires remains a key component in the diagnostic process in bowel endometriosis (21–23). The presence of specific GI symptoms may raise clinicians’ suspicion of intestinal involvement, prompting an expanded diagnostic approach for more accurate diagnosis and referral to specialized centers. Nevertheless, the relationship between symptomatology and the presence, localization and size of intestinal lesions remains controversial. Although several studies demonstrated a correlation between dyschezia and the presence and/or size of bowel lesions (15, 16, 24), other authors did not confirm these findings.

Egekvist et al. found no association between differences in rectosigmoid nodule size and symptom dynamics during conservative treatment (12). Similarly, Pashkunova et al. reported no correlation between lesion size and the severity of gastrointestinal dysfunction (14). Roman et al. described the phenomenon of “satellite lesions” (≤ 2 mm), which may not be visible on imaging but can be detected intraoperatively (11). Such microinfiltrations may impact symptom development or persistence independently of the main lesion’s size.

In our cohort the severity of dyschezia and the severity of abdominal pain and hematochezia were higher in patients with large rectal nodules (#Enzian C3), while no significant differences were observed between C1 and C2 lesions. In the subgroup of combined #Enzian C and FI lesions, the severity of hematochezia was higher, which is likely related to the fact that combined lesions are usually associated with larger rectal nodules (C2, C3). However, the frequency of hematochezia was only 8.9% of all cases, which limits its diagnostic value.

In combined intestinal endometriosis (#Enzian C and FI), the rectal nodule seems to be the main driver of dyschezia, as additional involvement of the FI compartment did not show any significant difference.

Our data showed that patients with additional intestinal lesions (#Enzian FI) are more likely to present with bloating than patients with rectal endometriosis alone. The correct diagnostic identification and respective surgical treatment of those additional bowel lesions in patients with rectal endometriosis, may potentially reduce persistent postsurgical bloating. Intestinal lesions may also remain asymptomatic or only cause mild discomfort (1, 8, 10). In our cohort, approximately 10% of patients with IE were considered mild or asymptomatic. A regular follow-up strategy by transvaginal ultrasound has been proposed in such cases, as bowel lesions in most patients do not progress over time (25, 26).

Intestinal endometriosis is usually associated with other endometriotic lesions and is rarely isolated. In our study, combined forms were diagnosed in 94.8% of cases, while isolated IE was observed in only 5.2% of cases (19). The potential impact of other localizations of deep endometriosis, such as retrocervical, vaginal (#Enzian A), lateral pelvic wall (#Enzian B), or adenomyosis (#Enzian FA), on symptom severity has also been discussed in the literature (14, 15, 17, 24). Involvement of these sites may play a significant role in the development of pain-related symptoms and GI dysfunction.

Pashkunova et al. concluded that additional involvement of #Enzian compartments A and/or B was associated with increased dyspareunia severity (14). Another retrospective study found no overall differences in symptom profiles across #Enzian types; however, severe dyschezia (VAS ≥ 5) correlated with #Enzian compartment C involvement, whereas dyspareunia correlated with adenomyosis (#Enzian FA) (24). Similarly, a study assessing reproducibility of the #Enzian classification via transvaginal ultrasound demonstrated associations between bowel symptoms and compartment B lesions (27).

Recent studies employing multidimensional analytical approaches have revealed distinct symptom clusters, particularly associating “severe pain” profiles with retrocervical and rectosigmoid involvement (28).

This study has several limitations. First, the cohort consisted of surgically treated women referred to a tertiary care center due to refractory pain, severe gastrointestinal symptoms, or/and infertility. This fact introduces selection bias and limits the generalizability of the findings to women with intestinal endometriosis managed conservatively. Second limitation is the lack of objective data regarding symptom duration and progression over time, as symptom assessment was based on patient-reported measures prior to surgery.

Third, intestinal endometriosis frequently coexisted with other forms of deep endometriosis, and the present analysis was not adjusted for concomitant non-intestinal lesions. Such associations,as discussed previously, may have influenced symptom severity in our cohort. However, the focus of the present study was on the overall symptom profile of women with intestinal endometriosis undergoing surgical treatment for endometriosis, that is particularly relevant in clinical practice. Another important limitation is the small size of subgroups of women with #Enzian FI involvement (*n* = 22) and combined #Enzian C and FI lesions (*n* = 11). Small groups and heterogeneity of FI locations may have limited the statistical power to detect significant differences. The absence of statistically significant associations in analyses should be interpreted with caution and does not exclude potential clinically relevant effects. Given the number of symptom outcomes and subgroup comparisons, the risk of type I error due to multiple testing cannot be excluded. Therefore, marginal *p*-values should be interpreted with caution, and the findings of exploratory analyses should be considered hypothesis-generating.

Additionally, the potential effect of hormonal treatment was not assessed in this cohort. Forty-four patients received hormonal therapy prior to surgical treatment. Hormonal therapy was discontinued following shared decision-making between the physician and the patient at least 3 months before surgical intervention. Although preoperative hormonal therapy was discontinued at least 3 months before surgery, its potential residual effects on symptom perception cannot be completely excluded.

Subjective symptom evaluation should remain an essential component of the comprehensive assessment of patients with endometriosis. Symptom evaluation may be useful to suspect IE and facilitate referral to specialized centers. However, the presence and severity of symptoms alone cannot confirm or exclude IE. Thus, diagnosis should be based on all available data, including imaging. Further research is needed to improve preoperative diagnostic accuracy of additional and multifocal intestinal lesions and optimize its management.

Ideally, further prospective trials are needed to analyze the role of multifocal intestinal lesions and their diagnostic challenges and effects on symptomatology in relation to other localizations and phenotypes of endometriosis. The estimation of bowel-related symptomatology remains crucial in order to decide whether respective surgical treatment is necessary and which surgical approach might be the most appropriate to reduce symptoms.

## 5 Conclusion

Large intestinal lesions (#Enzian C3) are associated with more severe dyschezia, abdominal pain, and hematochezia. The presence of additional intestinal lesions (#Enzian FI) is associated with increased severity of hematochezia and the risk of bloating.

## Data Availability

The raw data supporting the conclusions of this article will be made available by the authors, without undue reservation.
